# Predictors of Outcome and Hemorrhage in Patients Undergoing Endovascular Therapy with Solitaire Stent for Acute Ischemic Stroke

**DOI:** 10.1371/journal.pone.0144452

**Published:** 2015-12-07

**Authors:** Shaowei Jiang, Aihua Fei, Ya Peng, Jun Zhang, You-ran Lu, Hai-rong Wang, Miao Chen, Shuming Pan

**Affiliations:** 1 Emergency Department, Xinhua Hospital, Shanghai Jiao Tong University School of Medicine, Shanghai, 200092, China; 2 Cerebral Vascular Disease Center, The First People’s Hospital of Changzhou, Soochow University, Changzhou, 213003, China; 3 Department of Medical Imaging, The First People’s Hospital of Changzhou, Soochow University, Changzhou, 213003, China; Massachusetts General Hospital, UNITED STATES

## Abstract

**Background:**

Endovascular mechanical thrombectomy is emerging as a promising therapeutic approach for acute ischemic stroke and show some advantages. However, the data of predicting clinical outcome after thrombectomy with Solitaire retriever were limited. We attempt to identify prognostic factors of clinical outcome in patients with acute ischemic stroke undergoing thrombectomy with Solitaire retriever.

**Methods:**

We conducted a retrospective analysis of consecutive acute ischemic strokes cases treated between December 2010 and December2013 where the Solitaire stent retriever was used for acute ischemic stroke. We assessed the effect of selected demographic characteristics, clinical factors on poor outcome at 3 months (modified Rankin score 3–6), mortality at 3 months, and hemorrhage within 24 h (symptomatic and asymptomatic). Clinical, imaging and logistic variables were analyzed. A multivariate logistic regression analysis was used to identify variables influencing clinical outcome, based on discharge NIHSS score change and mRS at 3 months.

**Results:**

Eighty nine consecutive patients with acute ischemic stroke underwent mechanical thrombectomy. Multivariate analysis revealed that admission NIHSS score, Serum glucose and endovascular procedure duration were independently associated with clinical outcome. Sex, NIHSS score at admission, diabetes and time of operation were associated with sICH in 1 day. NIHSS score ≥20 (OR 9.38; 95% CI 2.41–36.50), onset to reperfusion >5 hours (OR 5.23; 95% CI1.34,20.41) and symptomatic intracranial hemorrhage (OR 10.19; 95% CI1.80,57.83) were potential predictive factors of mortality at 3 months.

**Conclusion:**

Multiple pre- and intra-procedural factors can be used to predict clinical outcome, symptomatic intracranial hemorrhage and mortality in acute ischemic stroke patients undergoing endovascular therapy. This knowledge is helpful for patients selection for endovascular mechanical thrombectomy.

## Introduction

Arterial reperfusion is consisdered as the main criterion of successful early management of acute ischemic stroke(AIS). Intravenous(IV) thrombolysis and endovascular thrombectomy are two major strategies to achieve recanalization. Although IV recombinant tissue plasminogen activator(rt-PA) is the approved treatment for AIS, the narrow time window and high number of exclusion criteria limite its applications[1,2]. Endovascular mechanical thrombectomy reaches higher rate of recanalization and good clinical outcome, has longer applying time window compared with IV thrombolysis[3,4]. Moreover, patients treated with new stent device, SOLITAIRE FR, shows higher revascularization rate and better neurological outcome compared with the earlier generation device (Merci Retriever, Stryker Neurovascular) [5,6]. Further more, recent studies indicate that rapid thrombectomy treatment after stroke onset resulted in higher reperfusion rate, better functional recovery and similar safety compared with IV rt-PA treatment[7–9].

Solitaire stent device provides us the fast and technically simple reperfusion resolution. However, limited data are available regarding predictors of outcome after thrombectomy with this third generation device[10]. It is important to identify criterias for patients likely to benefit from thrombectomy in order to improve selection and subsequently clinical prognosis. Therefore, we retrospectively evaluated the medical history and clinical data of 89 consecutive patients with symptomatic AIS treated with the Solitaire™ FR device (ev3/Covidien, Irvine, CA, USA) at our institution in a 35-month period. We attempted to identify notable factors predicting poor outcome, symptomatic intracranial hemorrhage(sICH) and death in patients with AIS undergoing mechanical thrombectomy with solitaire stent retriever.

## Patients and Methods

This retrospective study had been approved by Ethics Committee of the The First People’s Hospital of Changzhou, Soochow University. As the protocol used in this study is a method approved by the local Institutional Review Board, the requirement for individual patient consent or consent of their relatives was waived, and informations of patients were anonymized prior to analysis. We retrospectively analyzed the angiographic and clinical data of 89 consecutive patients with acute ischemic stroke due to large vessel occlusion who were treated with Solitaire FR stent either alone or in combination with application of thrombolytic drugs, balloon angioplasty or stent-assisted angioplasty in The First People’s Hospital of Changzhou, Soochow University between December 2010 and December 2013. The baseline NIHSS score, a clinical measure of neurologic deficit with a range of 0 (no deficit) to 42 (maximum possible deficit), was used to identify patients with a score of 10 or more, who have a greater than 80% likelihood of a major arterial occlusion[11]. The main inclusion criteria were: NIHSS score ≥10; treatment performed within 6 h from the onset of symptoms and no large hypodensity on CT or multimodal MRI; and occlusion of a major cerebral artery on the cranial CT, CTA or MRA. When vessel imaging was unavailable, NIHSS score ≥10, coma, hemiplegia, tetraparesis and aphasia were used as the proximal occlusion criteria.

Key exclusion criteria included uncontrolled hypertension, serious sensitivity to radiographic contrast agents, and CT or MRI evidence of intracranial hemorrhage or major ischemic infarction(acute ischemic change in more than a third of the middle cerebral artery territory or more than 100 mL of tissue in other territories)[5].

On admission, a stroke neurologist examined all patients clinically. Cranial CT or multimodal MRA were obtained prior to every intervention to confirm the diagnosis of large vessel occlusion and to rule out intra-cranial hemorrhage. Interventional treatment was initiated within 6 hours from onset of stroke symptoms.

The following data were collected: age, gender, cerebrovascular risk factors, baseline functional level prior to stroke onset (according to modified Rankin Scale, mRS), admission National Institutes of Health Stroke Scale (NIHSS) score, time of symptom onset. Technical details of the endovascular procedure that were collected included time from symptom onset to femoral artery puncture, Solitaire FR stent size, number of retrieval passes, and use of other intraarterial devices and pharmacologic agents.

Procedural factors that were captured included time to procedure from symptom onset, procedure duration, location of the vascular occlusion, presence of collateral support, and degree of recanalization based on the thrombolysis in cerebral infarction (TICI) scoring system. TICI grade 0 was defined as no perfusion; grade 1 was defined as perfusion past the initial obstruction but limited distal branch filling with little or slow distal perfusion; grade 2a was defined as perfusion of <1 of the vascular distribution of the occluded artery; grade 2b was defined as perfusion of greater than two thirds of the vascular distribution of the occluded artery; and grade 3 was defined as full perfusion with complete filling of all distal branches (some delay was accepted in the presence of proximal vasospasm or competitive collateral flow)[12]. Successful recanalization of the middle cerebral artery required reperfusion through all M1 and M2 segments. Successful recanalisation of internal carotid artery terminus lesions required reperfusion through the internal carotid artery and all M1 and M2 branches. Successful recanalization of a vertebral artery required reperfusion through both the target vertebral artery and the basilar artery.

Intracranial hemorrhagic transformations, were divided into clinically silent or symptomatic, and then classified into different categories. Hemorrhage was scored using the Pessin criteria and formalized in the ECASS trials (hemorrhagic infarction, types 1 and 2; parenchymal hematoma, types 1 and 2 [13–15]. Symptomatic intracranial hemorrhage was defined as any parenchymal hematoma subarachnoid hemorrhage, or intraventricular hemorrhage associated with a worsening of the NIHSS score by four or more within 24 h [5].

Stroke mechanism according to the TOAST classification, subtypes of acute ischemic stroke were defined at the 3-month follow-up as follows: cardioembolic, large vessel atherosclerosis, other (uncommon etiologies), or undetermined[16]. When no etiology was found or when two diagnoses were possible, the etiology was classified as undetermined.

Functional dependence was defined as a score of 3 to 6 on the modified Rankin Scale at 90 days. Functional independence was defined as a score of 0 to 2 on the modified Rankin Scale at 90 days.

### Statistical Methods

Quantitative variables were described as mean and standard deviation and qualitative data as number and percentage. SPSS for windows statistical software(Version 16.0; SPSS Inc., Chicago, IL, USA) was used for *t-TEST* and Chi-2 test analyses. Univariate logistic regression and multivariate (step wise logistic regressions, with enter and removal limits set at 0.10 and factors significant at *p* = 0.05 included) analyses were performed to determine factors associated with poor functional outcome at 3 months, mortality at 3 months and hemorrhage at 1 day. A *p* value<0.05 was considered significant. Odds-ratio(OR) and their 95% confidence intervals were calculated. Regression statistical tests were performed using SAS version 9.4 statistical software (SAS Inc., Cary, NC, USA).

## Results

### Population baseline

Fifty five men and 34 women were retrospective included with a mean age of 63 (range 21–85) years and a mean initial NIHSS score of 19 (range 10–34). Of 89 patients in this study, 44 patients suffered atrial fibrillation, 13 patients surffered diabetes mellitus and 26 patients with hypertension. Causes of stroke were cardiogenic embolism in 42 patients(47.2%), large-artery atherosclerosis in 39 patients(43.8%). Other or undetermined reasons for stroke was happended in 5 (5.6%) and 3(3.4%) patients respectively. The occlusion involved the anterior circulation in 81 (91%) patients, posterior circulation in 7 patients and anterior plus posterior circulation in one patient. There were 17 patients(19.1%) with arterial stenosis and 4 patients (5.6%) with arterial dissection. Detailed baseline and clinical characteristics of these patients were shown in Table 1.

**Table 1:** Basline of clinical and neuroimaging characteristics of population.

Demographics	Value(n, % Unless Otherwise Specified)
Age (years), mean ± SD	63.12±13.98(21–85)
Sex (male: female)—no.(%)	(55:34)(61.80%:38.20%)
NIHSS score on admission, mean ± SD	19.17±4.64(10–34)
NIHSS score on admission, median	19
NIHSS score at discharge	10.52±11.09(0–42)
Cerebrovascular risk factors	
Atrial fibrillation	44(49.44%)
Coronary artery disease or myocardial infarction	
Diabetes mellitus	13(14.61%)
Serum glucose, mmol/l mean±(SD) (range)	7.97±3.57(3.9–20)
Hypertension	26 (47.3%)
Blood Pressure, mmHg mean±(SD) (range)	
Systolic	136.48±24.34 (87–192)
Diastolic	80.39±13.86 (52–112)
TG>2.02mmol/l—no.(%)	5(9.1)
Triacylglycerol, mmol/l mean±(SD) (range)	1.79±0.98 (0.68–4.71)
TC>5.7mmol/l—no.(%)	13(23.6)
Cholesterol, mmol/l mean±(SD) (range)	4.40±0.89 (2.19–6.89)
Stroke cause on day 7—no. (%)	
Cardiogenic embolism	42(47.19%)
Large-artery atherosclerosis	39(43.82%)
Other	5(5.62%)
Undetermined	3(3.37%)
Distribution of vascular occlusion, stenosis and dissection	
Anterior circulation	81(91.01%)
MCA M1/M2	62(69.67%)
ACA A1/A2	2(2.25%)
ICA	4(4.49%)
Tandem occlusion (ICA1 MCA M1/M2)	13(14.61%)
Posterior circulation	7(7.87%)
Basilar	4(4.49%)
vertebral	1(1.12%)
Tandem occlusion(vertebral+ Basilar, Basilar+ PCA P1/P2)	2(2.25%)
Anterior and posterior circulation	1(1.12%)
Site of Stenosis	17(19.10)
MCA—no.(%)	13(14.61)
ICA—no.(%)	2(2.25)
BA—no.(%)	2(2.25)
Site of Dissection	5(5.62)
ICA—no.(%)	4(4.49)
VA—no.(%)	1(1.12)

### Thrombectomy data and results

The mean time between symptom onset to operation was 171 min (range 60–356), and mean value of time from needle to recanalization was 115 min (range 49–420). The total time between onset to recanalization ranged from 120 min to 660 min, with a mean value of 285 min. Mean passes of the thrombectomy device used on each patient were 2.19 (range 1–6). Thirty two patients(36.0%) only received Solitaire stent treatment, 25 patients were treated with combination of stent and urokinase, 27(30.0%) and 2(2.2%) patients used Solitaire FR together with balloon and aspiration device(Penumbra) respectively. Decompressive craniectomy was applied on 12 patients(13.5%), and 28 patients (31.5%) suffered stent-assisted angioplasty. More details were showed in Table 2.

**Table 2:** Data of mechanical thrombectomy with Solitaire.

Demographics	Value(n, % Unless Otherwise Specified)
Onset to needle,min mean±(SD)(range)	170.64 ±67.73(60–356)
Door to needle,min mean±(SD)(range)	69.2±32.44(30–180)
Needle to recanalization,min mean±(SD)(range)	114.51±63.65(49–420)
Onset to recanalization,min mean±(SD)(range)	285.15±94.23(120–660)
Number of passes with Solitaire FR mean±(SD)(range)	2.19±1.20(1–6)
1	33(37.08%)
2	21(23.60%)
3	23(25.84%)
>3	12(13.48%)
Multimodal endovascular therapy	
Solitaire FR only	32(35.96%)
IA urokinase—no.(%)	25(28.09%)
Dose of urokinase 10,000 IU mean±(SD)(range)	274194±123741(100000–550000)
Balloon—no.(%)	27(30.3%)
Stent-assisted angioplasty—no.(%)	28(31.46%)
Decompressive craniectomy—no.(%)	12(13.48%)
Aspiration thrombectomy with Penumbra	2(2.24%)

### Clinical results

As shown in Table 3, total recanalization rate was achieved in 67.4%, with TICI3 rate of 39.3% and TICI2b rate 28.1%. Clinical efficacy (mRS 0–2) at 3 months was achieved for 41.6% (37/89). Sixty-eight (76.4%) patients showed improvement of clinical symptoms (NIHSS≥4) at discharge, with 14 patients (15.7%) complete recovery. All patients underwent a follow-up CT scan within 1 day when neurological status worsened. Infarction hemorrhagic transformation occurred in 42 (47.2%), with the the following subtypes: 10 (11.24%) asymptomatic hemorrhagic infarction type (HI) 1, 13 (14.6%) asymptomatic HI2, 4 (4.5%) asymptomatic parenchymal hemorrhage type (PH) 1, 4 (4.5%) asymptomatic PH2, 11 (12.4%) symptomatic intracranial hemorrhage (sIHC).

**Table 3:** Outcome after endovascular thrombectomy treatment.

Demographics	Value(n, % Unless Otherwise Specified)
TICI recanalization	
0	6(6.74%)
1	5(5.62%)
2a	18(20.22%)
2b	25(28.09%)
3	35(39.33%)
Success of recanalization—no.(%)	78(87.64)
Complete recanalization (TICI 3)—no.(%)	35(39.33)
Partial recanalization (TICI 2a/2b)—no.(%)	43(48.31)
Recanalization failure (TICI 0/1)—no.(%)	11(12.36)
Improvement of clinical symptom—no.(%)(NIHSS ≥4)	68(51.96)
Complete recovery (NIHSS = 0)—no.(%)	14(15.73)
mRS ≤ 2 at 90 days—no.(%)	37 (41.57)
Infarction Haemorrhagic Transformation—no.(%)	42(47.2%)
Asymptomatic HI-1—no.(%)	10(11.24%)
HI-2—no.(%)	13(14.61%)
PH-1—no.(%)	4(4.49%)
PH-2—no.(%)	4(4.49%)
Symptomatic PH-2—no.(%)	7(7.87%)
IVH—no.(%)	4(4.49%)

### Prognostic factors for clinical outcome

Only three independent prognostic factors for clinical outcome were identified under multivariate logistic regression analysis, while nine factors showed significant association with functional recovery on univariate analysis (Table 4). Baseline NIHSS score (multivariate analysis OR 5.25, 95% CI 1.66–16.63), serum glucose (multivariate analysis OR 1.31, 95% CI 1.06–1.63) and time from needle to recanalization (multivariate analysis OR 2.97, 95% CI 1.00–8.83) were significantly associated with outcomes in both univariate and multivariate regression analysis. Additionally, sex (OR 4.23, 95% CI 1.63–10.98), 7th NIHSS score (OR 1.82, 95% CI 1.38–2.39), diabetes(OR 10.80, 1.34–87.23), blood pressure (systolic pressure OR 1.02, 95% CI 1.00–1.03; diastolic pressure OR 1.03, 95% CI 1.00–1.07) and atrial fibrillation (OR 2.72, 95% CI 1.14–6.52) showed correlationship with 3 month outcome only on univariate analysis (Table 4).

**Table 4:** Comparison between patients who had favorable and unfavorable outcome at 3 months.

Factors	MRS≤2	MRS≥3	Univariate analysis OR(95%CI)	Multivariate analysis OR(95%CI)
Sex				
Male	29(78.38)	24(46.15)	1.00	1.00
Female	8(21.62)	28(53.85)	4.23(1.63,10.98)	2.89(0.92,9.11)
NIHSS score on admission				
<20	29(78.38)	22(42.31)	1.00	1.00
≥20	8(21.62)	30(57.69)	4.94(1.90,12.87)	5.25(1.66,16.63)
NIHSS 7,mean±SD	2.49±2.57	16.23±11.29	1.82(1.38,2.39)	–
Diabete				
No	36(97.30)	40(76.92)	1.00	–
Yes	1(2.70)	12(23.08)	10.80(1.34,87.23)	–
Serum glucose,mean±SD	6.53±2.38	9.43±4.41	1.37(1.13,1.66)	1.31(1.06,1.63)
Systolic,mean±SD	137.19±22.96	149.62±29.19	1.02(1.00,1.03)	-
Diastolic,mean±SD	80.19±12.28	86.13±14.20	1.03(1.00,1.07)	-
Atrial fibrillation				
No	24(64.86)	21(40.38)	1.00	–
Yes	13(35.14)	31(59.62)	2.72(1.14,6.52)	–
Needle to recanalizatio,mean±SD	96.70±47.72	127.17±70.63	1.01(1.00,1.02)	2.97(1.00,8.83)
Onset to recanalization				
<5H	24(64.86)	23(44.23)	1.00	–
≥5H	13(35.14)	29(55.77)	2.33(0.98,5.55)	–

### Prognostic factors for sICH

Female (OR 8.50, 95% CI 1.71–42.17), increased admission (OR 7.60, 95% CI 1.54–37.64) and 7th day (OR 1.09, 95% CI 1.03–1.15) NIHSS scores, diabetes (OR 7.29, 95% CI 1.81–29.40), serum glucose (OR 1.25, 95% CI 1.07–1.47), and increased time from needle to recanalization (OR 1.01, 95% CI 1.00–1.01) were associated with sICH in the univariate analysis. On the multivariates analysis, Female (OR 10.34, 95% CI 1.34–79.59), increased admission NIHSS scores (OR 9.73, 95% CI 1.34–70.69), diabetes (OR 7.34, 95% CI 1.32–40.84) and increased time from needle to recanalization (OR 1.01, 95% CI 1.00–1.02) remained significantly associated with sICH. The final multivariable model is shown in Table 5.

**Table 5:** Analysis of potential factors predicting symptomatic intracranial hemorrhage (sICH) at day 1.

Factors	No sICH	sICH	Univariate analysis OR(95%CI)	Multivariate analysis OR(95%CI)
Sex				
Male	51(65.38)	2(18.18)	1	1
Female	27(34.62)	9(81.82)	8.50(1.71,42.17)	10.34(1.34,79.59)
NIHSS score on admission				
<20	49(62.82)	2(18.18)	1	1
≥20	29(37.18)	9(81.82)	7.60(1.54,37.64)	9.73(1.34,70.69)
NIHSS 7,mean±SD	8.92±10.57	21.82±7.87	1.09(1.03,1.15)	–
Diabete				
No	70(89.74)	6(54.55)	1	1
Yes	8(10.26)	5(45.45)	7.29(1.81,29.40)	7.34(1.32,40.84)
Serum glucose,mean±SD	7.66±3.62	12.32±4.10	1.25(1.07,1.47)	–
Needle to recanalization,mean±SD	110.92±54.16	139.91±110.66	1.01(1.00,1.01)	1.01(1.00,1.02)
Onset to recanalization				
<5H	41(52.56)	6(54.55)	1	–
≥5H	37(47.44)	5(45.45)	0.92(0.26,3.28)	–

### Prognostics factors for mortality

Of 89 cases, 21 patients (23.6%) were deceased at 3 months, 13 patients(14.6%) had malignant cerebral edema, and 8 patients (9.0%) had sICH. Both univariate and multivariate logistic regression analysis were performed to identify mortality prognostic factors. Symptomatic ICH (OR 10.19, 95% CI 1.80–57.83) and admission NIHSS score (OR 9.38, 95% CI 2.41–36.50) were important prognositc factors of death. Interestingly, the time of symptoms onset to recanalization (OR 5.23, 95% CI 1.34–20.41), insteading of needle to recanalization (univariate analysis OR 1.01, 95% CI 1.00–1.02), showed signicant association with mortality under multivariate analysis. Moreover, other six independent prognostic factors of mortality, including increased age (≥ 70 OR 3.59, 95% CI 1.01–12.73) and 7th day NIHSS scores (OR 1.30, 95% CI 1.16–1.44), higher serum glucose (OR 1.17, 95% CI 1.03–1.33), multiple affected hemisphere basline image (OR 4.65, 95% CI 1.13–19.21), 2 passes with Solitaire FR (OR 4.46, 95% CI 1.14–17.50) and needle to recanalization (OR 1.01, 95% CI 1.00–1.02), also were identified by univariate logistic regression analysis. Results of the univariate and multivariate analysis of potential factors predictive of mortality at 3 months are detailed in Table 6.

**Table 6:** Analysis of potential factors predictive of mortality at 3 months.

Factors	Death	Alive	Univariate analysis OR(95%CI)	Multivariate analysis OR(95%CI)
Age				
≤60	4(19.05)	30(44.12)	1	–
60–70	6(28.57)	15(22.06)	3.00(0.73,12.27)	–
>70	11(52.38)	23(33.82)	3.59(1.01,12.73)	–
NIHSS score on admission				
<20	4(19.05)	47(69.12)	1	1
≥20	17(80.95)	21(30.88)	9.51(2.85,31.73)	9.38(2.41,36.50)
NIHSS 7,mean±SD	25.81±11.09	5.79±5.38	1.30(1.16,1.44)	–
Serum glucose,mean±SD	10.26±4.33	7.58±3.63	1.17(1.03,1.33)	–
Affected hemisphere on baseline imaging				
Left_hemisphere	8(38.10)	31(45.59)	1	–
Right_hemisphere	7(33.33)	32(47.06)	0.85(0.27,2.62)	–
Multiple	6(28.57)	5(7.35)	4.65(1.13,19.21)	–
Number of passes with Solitaire FR				
1	4(19.05)	29(42.65)	1	–
2	8(38.10)	13(19.12)	4.46(1.14,17.50)	–
3	6(28.57)	17(25.00)	2.56(0.63,10.37)	–
>3	3(14.29)	9(13.24)	2.42(0.45,12.88)	–
Needle to recanalization,mean±SD	152.1±80.1	102.9±53.2	1.01(1.00,1.02)	–
Onset to recanalization				
<5H	7(33.33)	40(58.82)	1	1
≥5H	14(66.67)	28(41.18)	2.86(1.02,7.99)	5.23(1.34,20.41)
Infarction Haemorrhagic Transformation				
NO_sICH	13(61.90)	65(95.59)	1	1
sICH	8(38.10)	3(4.41)	13.33(3.11,57.09)	10.19(1.80,57.83)

## Discussion

According to the newest American Heart Association/American Stroke Association (AHA/ASA) guidelines for early management of patients with AIS, endovascular thrombectomy with the third generation stent has been proved to an important strategy to manage AIS[17]. Here, we presented our single-center retrospective study about Chinese AIS patients treated with mechanical thrombectomy. We focus on identifying prognostic factors for not only clinical outcomes but also sICH after endovascular treatment with Solitaire. At 3 months, the independent prognostic factors for clinical outcome were NIHSS score on admission, serum glucose and time from needle to recanalization. Prognostics for mortality after 3 month were NIHSS score on admission, time from symptom onset to recanalization and sICH. Sex, NIHSS score on admission, diabetes and time from needle to recanalization were associated with sICH in 1 day.

### Comparison with other Solitaire studies

Here, we showed our endovascular treatment data from single center. In comparison with other thrombectomy studies, little lower recanalization rate (67.4%) was observed, but clinical efficacy (41.6%) was similar with these trails [5,7,18–20]. With respect to previous study, a slightly higher sICH rate (12.4%) and mortality (23.6%) probably were due to the different selection criteria in terms of admission NIHSS score or other variants [5,8,20,21].

The predictors of AIS patients treated with Solitaire stent are variable in different studies. In Soize’s study, the angiography data, ASPECT score, was the important predictor for clinical outcome, mortality and sICH, and thrombus length, recanalization and endovascular procedure time were associated with clinical outcome[22]. Another study about French AIS patients showed the prognostic factors for outcome were age, sex, site of occlusion and initial NIHSS score[19]. Recanalization time and FLAIR negativity were associated with clinical outcome in Raoult’s study, while Costalat et al found hyperglycemia and baseline NIHSS score could predict the clinical outcome [10,21]. In our study, we found initial NIHSS score, hyperglycemia and endovascular procedure time associated with clinical outcome, but no angiography data prognostic factors were observed. The different studies concluded different predictors might be caused by patient population difference, or whole treatment procedure variance, or some other statistic factors such as limited patients.

### NIHSS score at admission

NIHSS score is a quick and relatively simple guide to estimate the extent and the severity of stroke, and the newest guideline point out that NIHSS>6 is an important patient selection criteria for thrombectomy treatment[17,23]. In this study, baseline NIHSS score of all the stroke patients were above 10. As NIHSS score is the most powerful predictor for long-term outcome of patients with AIS[24], we also found it associated with clinical outcome, mortality and sICH in the present cohort. Lower NIHSS score (NIHSS<20) showed significant association with favor outcomes (OR 5.25). Patients with admission NHISS score ≥ 20 only showed 21.1% (8/38) clinical efficacy, while functional recovery rate was 56.9% (29/51) in less severe stroke patients (NIHSS<20). However, it does not mean mechanical thrombectomy is not recommended on patients with high score of admission NIHSS, and rapid arterial reperfusion still is an effective strategy on severe stroke patients [25]. Actually, endovascular treatment for patients suffered moderate or severe stroke (NIHSSS>15 or NIHSSS≥ 20) showed great potential versus intravenous thrombolysis[26–28].

### Sex

Sex is a controversial factor predicting AIS outcomes. In our study, sex appeared correlated with functional outcome under univariate regression analysis, however, did not significantly associate with clinical outcome on multivariates analysis. This is in accordance with the previous result of association analysis on sex and thrombolysis outcome[29,30]. In Hametner’s study, sex was no longer the prognostic factor even on the univariates analysis after matching other factors (such as age, NIHSS score et al.) for the female and male pateints[29]. Interestingly, age and prevalence rate of atrial fibrillation were significantly higher in female patients than males in our cohort (Table 7). Age was considered as prognostic factors in other studies [21,31], though it did not associate with functional outcomes in the present study. Furthermore, atrial fibrillation was an independent prognostic factor in our univariate regression analysis (Table 4), and female atrial fibrillation patients with stroke usually were suggested worse outcome [32,33]. Consequently, that sex was considerd as prognostic factors for clinical outcome might be contributed from the difference in age and incidence of atrial fibrillation.

**Table 7:** Factors distributed between female and male.

	Male	Female	Test	*P* value
Age (mean±SD)	59.5 ± 14.5	68.4 ± 11.5	*T*-test	0.003**
NIHSS score on admission(mean±SD)	18.4 ± 5.5	19.8 ± 4.5	*T*-test	0.223
IA urokinase				
Yes	20	11	Chi-2	0.485
No	33	25		
Dose of urokinase (x 1,000 IU) mean±(SD)	10.47 ± 15.91	8.19 ± 13.64	*T*-test	0.485
Diabetes				
Yes	5	8	Chi-2	0.094
No	48	28		
Serum glucose(mean±SD)	7.80 ± 4.19	8.82 ±3.48	*T*-test	0.240
Hypertension				
Yes	31	20	Chi-2	0.784
No	22	16		
Atrial fibrillation				
Yes	20	24	Chi-2	0.007**
No	33	12		
Onset to needle (mean±SD)	175.5 ± 64.0	170.0 ± 86.2	*T*-test	0.735
Needle to recanalization (mean±SD)	117.0 ± 71.9	110.2 ± 51.2	*T*-test	0.633
Onset to recananlization (mean±SD)	290.9 ± 101.8	280.2 ± 88.3	*T*-test	0.617

** indicated significant difference.

Although sex was not the prognostic factor for clinical outcome, female predicted higher frequency of sICH both before and after adjusted in our cohort. On the coarsened exact matching study, sex was obviously associated with sICH in stroke thrombolysis[29]. But, whether dose of rt-PA effected the association between sex and sICH was still not clear in their research[29]. As the frequency and dose of urokinase administered to stoke patients did not show significant difference between male and female (Table 7), the effect of thrombolytic agents was excluded in our endovascular thrombectomy study. However, we could not eliminate the interference of imbalanced age and atrial fibrillation distribution on the correlation of sex and sICH.

### Serum glucose and diabetes

Hyperglycemia is a common feature of acute stroke, and admission hyperglycemia in ischemic stroke patients often associated with worse prognosis and higher mortality[34–36]. Hyperglycemia was considered as an epiphenomenon of acute stress response, and might aggravate cerebral damage[37–39]. Hyperglycemia also impaired recanalization and decreased reperfusion rate in patients treated with intravenous thrombolysis[40]. Although the recanalization rate was in a relatively high level and reperfusion time was not affected by hyperglycemia in our study, we found blood glucose was helpful to predict the clinical outcome. Some other negative effects of hyperglycemia, such as increased reperfusion injury, might still lead to bad outcomes.

It is inconclusive whether ischemic stroke patients with diabetes have worse functional recovery till now[40]. Therefore, it was not surprised that diabetes was not associated with clinical outcome in our multivariates analysis, even if diabetes predicted worse outcome under univariate regression analysis (Table 4). This result was consistent with some recent thrombectomy trials[10,19,21,41]. On the other hand, diabetes is a condition of accelerated vascular aging and a risk factor for intracerebral hemorrhage[42,43]. We presented more than 7 fold risk of sICH in patients with diabetes compared with non-diabetes group both in univariant analysis and multivariants models. In accordance with our finding, we emphasize that more attention should be paid to control sICH when endovascular thrombectomy was applied on patients with diabetes.

### Time of recanalization

Recanalization is helpful to prevent infarct growth and decreased delay of reperfusion time as possible is emphasized in the newest guideline for AIS patients management[17]. Recent multicenter study with stent retriever also revealed faster procedure time and shorter treatment delays can lead to better clinical outcomes[25,42]. Reducing delay from image to groin puncture time and shortering endovascular procedure duration for ischemic stroke therapy was likely associated with improved clinical outcome[44]. Correspondingly, the shorter time from needle to recanalization predicted independent functional outcomes and lower sICH rate in our study. It indicated the further potential to improve the endovascular procedure efficiency and perhaps achieve better clinical benefits.

Time from symptoms onset ro recanalization also is identified as an important mortality prognostic factor previously[8,25,45]. Not surprisingly, we found time from onset to reperfusion time beyond 5 hours was associated with mortality. The longer time of cerebral ischemic, the severer damage to brain happended. Earlier to hospital, faster determine time and more efficient operation procedure are important to reducing mortality.

Actually, to get better recanalization, we use some other methods, such as balloon angioplasty, stent-assisted angioplasty and injecting urokinase, et al, combined with Solitaire stent during thrombectomy procedure (Table 2). This multimode operation improved the thrombectomy and reperfusion efficiency.

### sICH, clinical outcome and mortality

Symptomatic intracranial hemorrhage often leads to bad outcome[46], however, it did not show significant association with clinical efficacy at 3 months, even in univariant analysis. We proposed that thrombectomy treatment or some other reasons might be benefit for recovery of patients suffered sICH, although hemorrhage made symptomatic more severe temporarily. Nevertheless, sICH increased the odds (more than 10 fold) of death in our regression model. This warn us sICH still is a severe complication and we should keep enough coping strategies when thrombectomy proceeded.

In summary, this retrospective study on AIS patients underwent endovascular thrombectomy not only point out some pre- and intra-procedural factors for predicting clinical outcome, sICH and mortality, but also provide some new proofs which are useful for interpreting the controversial prognostic factors (gender, diabetes) of sICH. These knowledges are important when obtaining an informed consent and conveying expectations regarding procedure outcomes, and might be helpful for improving our management of AIS patients.

Our study has several limitations. Limitations of the present study include the retrospective and single-center design. Nevertheless, we had enough statistical power to identify the important predictive factors within our series. Further analyses are needed to determine its importance in the selection of patients for mechanical thrombectomy.

This study is supported by 2013–2014 National Clinical Key Speciality Construction Project, and Science and Technology Commission of Shanghai Municipality grants 13ZR1426500
